# Synaptic remodeling of GluA1 and GluA2 expression in the nucleus accumbens promotes susceptibility to cognitive deficits concomitant with downstream GSK3*β* mediated neurotoxicity in female mice during abstinence from voluntary oral methamphetamine

**DOI:** 10.1016/j.addicn.2023.100112

**Published:** 2023-06-16

**Authors:** Nicoletta Memos, Jorge A. Avila, Edgar Rodriguez, Peter A. Serrano

**Affiliations:** a Department of Psychology, Hunter College, City University of New York, New York, NY, 10065, USA; b The Graduate Center of CUNY, New York, NY, 10016, USA; c Undergraduate Research Center – Sciences, University of California, Los Angeles, CA 90095, USA

**Keywords:** Hippocampus, Nucleus accumbens, Sex differences, AMPA receptors, Methamphetamine addiction

## Abstract

Stimulant-use disorders can present with long-term cognitive and mental health deficits. Little is known about the underlying molecular mechanisms perpetuating sex differences in cognitive and behavioral deficits in preclinical models of addiction to stimulants such as methamphetamine (MA). The current study investigated the neurochemical shifts underlying sex disparities in MA-induced working memory deficits and an addictive phenotype following abstinence from chronic MA abuse. We used our previously reported mouse model of voluntary oral methamphetamine administration (VOMA) consisting of an acquisition phase (days 1–14) characterized by escalating doses of MA and a binge phase (days 14–28) characterized by static doses. Female VOMA mice exhibited sustained MA consumption during the binge phase, demonstrating sex-specific vulnerabilities to the maintenance of MA addiction. The 8-arm radial maze was used to test spatial working memory performance following abstinence from VOMA. Results indicate working memory deficits correlated to higher MA consumption in females only. Hippocampal and accumbal tissue were collected and analyzed by immunoblotting. Female VOMA mice had decreased GluA1, but not GluA2, in the hippocampus, which may perpetuate synaptic destabilization and working memory deficits. Female-specific increases in GluA1 and p-GSK3*β* expression in accumbal tissue suggest vulnerability toward abstinence-induced drug craving and heightened downstream neurotoxicity. Our study reveals female-specific neurochemical shifts in hippocampal and accumbal AMPA receptor signaling following abstinence from chronic MA consumption that may perpetuate female susceptibility to MA-induced cognitive deficits. These data demonstrate a novel molecular pathway that would exacerbate memory deficits and perpetuate an addictive phenotype in female populations following MA abuse.

## Introduction

Substance use disorder (SUD) populations display a multitude of cognitive deficits following drug use including impaired decision making, attention, inhibition, and the regulation of motivational processes [1]. Specifically, women experience distinct forms and levels of mental health deficits as a consequence of substance misuse and are reported to have higher incidence rates of anxiety, depression, and suicidal thoughts compared to men [2]. Methamphetamine (MA) misuse is known to produce long-term neurotoxicity in brain regions responsible for memory processing, specifically the hippocampus which has been shown to undergo neurodegeneration [3–6]. Clinical studies show that women consume more MA during binge sessions of MA abuse, exhibit increased susceptibility to acquisition, maintenance, and relapse of MA use following withdrawal, and have greater downstream neurological deficits compared to men [3–10]. Additionally, adolescent female MA use in the United States has increased over time nationally and is associated with behavioral and neurological deficits [11], highlighting adolescent females as a susceptible population to the mental health risks of MA abuse. It is imperative to determine the underlying molecular mechanisms driving these sex-dependent differential patterns of abuse in female users to generate targeted intervention.

Rodent models of SUDs have identified sex differences in self-administration rates and escalation of MA use between male and female rats, highlighting sex differences throughout the different phases of addiction [12,13]. Previous studies indicate that female rats self-administer more MA, escalate their intake of MA faster, and display enhanced drug-seeking behavior compared to males [14–16]. However, the molecular mechanisms promoting these sex differences are poorly understood [16–20]. Furthermore, recent studies have identified sexual disparities in basal levels of molecular markers such as prodynorphin, kappa opioid receptor (*κ*OR), and corticotrophin-releasing hormone in brain regions involved in the incubation of MA craving and perpetuating an addictive phenotype following self-administration of MA [21–23]. Sexual dimorphisms in the dopaminergic molecular signaling cascades underlying female vulnerability to cocaine have also been reported in mice [24]. Previously, we identified sex-specific alterations in hippocampal protein kinase M zeta (PKM*ζ*) and *κ*OR expression that promoted female-specific working memory deficits and vulnerability towards the dysphoric effects of MA during withdrawal [25]. Investigation of *α*-amino-3-hydroxy-5-methyl-4-isoxazolepropionic acid receptor (AMPAR) expression downstream of PKM*ζ* activation may aid in elucidating signaling cascades involved in perpetuating sex specific behavioral deficits. To understand the molecular mechanisms that may be perpetuating increased female sensitivity to MA, we examined two brain regions which are essential in promoting the deficits observed in female MA users. We examined neurochemical signaling cascades in the hippocampus that underly working memory deficits and the nucleus accumbens (NAc) for the relationship between reward circuitry, drug-seeking behavior, and synaptic plasticity. Rodent studies have demonstrated that the NAc is implicated in spatial memory, memory consolidation, and synaptic plasticity through AMPAR dependent signaling in a variety of behavioral paradigms [26–29]. The NAc is also known to have projections and neural circuitry connections to critical brain regions involved in learning and memory including the hippocampus, dorsal striatum, prefrontal cortex, and amygdala [26, 30–32]. Repeated exposure to amphetamines in rats has been shown to promote synaptic plasticity changes resulting in deficits in spatial learning and memory tasks accompanied by underlying changes in spine density of medium spiny neurons (MSNs), suggesting further implications in downstream drug taking behavior [33] We hypothesize that MA-induced shifts in female hippocampal GluA1 expression following withdrawal from chronic MA is associated with working memory deficits. We also hypothesize that shifts in accumbal GluA1 expression may underlie synaptic remodeling implicated in the incubation of craving during withdrawal and produce increased susceptibility to drug-seeking behaviors in addition to exacerbating MA-induced memory deficits following a period of prolonged abstinence.

Clinical reports indicate that brain regions responsible for drug-seeking behaviors, information processing, and memory formation are severely deteriorated following chronic MA abuse [34–38]. Following three to 15 weeks of abstinence, adult MA users demonstrate significant persistent memory deficits on memory performance tasks [20]. Rodent studies have identified similar deficits in memory performance following MA abuse [14,39]. However, the AMPAR signaling pathways that drive synaptic plasticity have not been fully investigated in MA-induced memory deficits. GluA1 and GluA2 are AMPARs known to be involved in synaptic plasticity processes with direct implications in learning and memory [40,41]. AMPAR trafficking is modulated by PKM*ζ* to sustain LTP processes [42,43] and conversly by glycogen synthase kinase 3*β* (GSK3*β*) to promote LTD [44]. Previously we demonstrated persistent working memory deficits following withdrawal from chronic voluntary MA administration (VOMA) in male mice associated with decreased PKM*ζ* and GluA2 expression in the hippocampus [45]. We hypothesize that shifts in hippocampal GluA1 following withdrawal from MA promotes synaptic destabilization and working memory deficits in a sex-specific manner. In rodent SUD studies, calcium-permeable (CP) GluA1 and calcium impermeable (CI) GluA2 signaling in the NAc are both associated with the incubation of drug craving [46–49]. CP-GluA1, but not CI-GluA2, is upregulated following prolonged withdrawal from both cocaine [46–48] and MA administration [49,50] in rodent self-administration models where active nose pokes or lever presses result in drug infusions. Furthermore, administration of a selective CP-AMPAR antagonist, Naspm, directly in the NAc inhibits cocaine seeking behavior in rat self-administration models [46], highlighting the implication of these AMPARs in craving and drug seeking behavior following with-drawal. Enhanced AMPAR signaling following repeated cocaine administration and increased CP-GluA1 expression promotes increased spine density of MSNs in the NAc, predicted to underlie drug seeking and craving behavior [51–53]. Although accumbal modulations in AMPARs have been implicated in a variety of addictive behaviors including the incubation of craving and drug seeking behaviors utilizing self-administration models of SUDs as well as behavioral paradigms such as conditioned place preference and sensitization models [54], few studies have addressed the neurochemical shifts in AMPAR signaling following spatial memory assessments. Our study aims to progress this work forward by focusing on a novel voluntary self-administration model of MA use and examining the neurochemical modulations in AMPAR expression following short term working memory assessments on the radial-8 arm maze that remain underexplored in current animal models of SUDs. Based on the current literature, we hypothesize that increased GluA1 expression in the NAc of female mice following abstinence from chronic MA may promote the sensitization of neuronal responses to calcium and glutamate release. Calcium mediated neuronal sensitization may result in female-specific susceptibility to MA-induced drug-craving and drug seeking. Increased susceptibility to drug craving and drug seeking may then exacerbate female memory deficits due to calcium dysregulation and synaptic destabilization.

MA is known to produce neurotoxic excitotoxicity through glutamate signaling with downstream activation of cell death and inflammatory pathways [55,56]. GSK3*β* signaling is heavily implicated in neuronal apoptosis, downstream neuroinflammatory signaling cascades as well as synaptic plasticity processes [41,44,46–48,57]. Little is known regarding the role of GSK3*β* regulation of calcium and glutamate-dependent synaptic plasticity in both clinical MA abuse and rodent models of MA abuse. One study reported female-specific increases in striatal GSK3*β* expression and upstream phosphorylated serine-threonine kinase (Akt) and extracellular signal-related kinase $\frac{1}{2}$ (ERK1/2) expression following chronic MA administration [17], hinting at potential female-specific neuroprotection from the detrimental effects of MA. However, there is a lack of research on how GSK3*β* regulates AMPAR signaling cascades to affect synaptic plasticity and reward circuitry to promote downstream behavioral deficits following abstinence from MA. The investigation of how these signaling cascades are implicated in MA abuse is crucial to understand the neurochemical shifts driving sex differences in detrimental MA-induced memory and behavioral deficits. We hypothesize that changes in hippocampal p-GSK3*β* promotes a subsequent shift in calcium signaling at the synapse promoting LTD and associated AMPAR endocytosis, exacerbating synaptic destabilization with concomitant poor performance on a hippocampal spatial memory task. We hypothesize that female-specific dysregulated p-GSK3*β* signaling promotes increased CP-AMPAR in the NAc in response to dysregulated calcium and glutamate release. Enhanced CP-AMPAR signaling may promote susceptibility to MA-induced drug craving and drug seeking.

The current study uses a voluntary oral MA Administration model of stimulant use disorders, VOMA, that is known to produce hippocampal dependent deficits in short-term working memory in male mice [45]. We focus on the underlying AMPAR signaling cascades and GSK3*β* synaptic plasticity regulation in adolescent male and female mice to identify sex differences in the hippocampus that may underly cognitive deficits following MA abuse. We focus on these same signaling cascades in the NAc which may promote female specific susceptibility to drug seeking and relapse following withdrawal from MA abuse with downstream enhanced vulnerability to persistent MA-induced memory deficits.

## Materials and methods

### Animals

Experimental conditions, housing, and drug administration procedures were reviewed and approved by the Institutional Animal Care and Use Committee (IACUC) of Hunter College. Male and female C57/Bl6 mice were purchased from Taconic Biosciences (Rensselaer, NY) and were received at the Hunter College Animal facility at 6 weeks of age. Mice were kept on a 12 h/12 h light/dark cycle from 7:30 h to 19:30 h, housed individually upon arrival, and fed ad libitum for 1 week prior to handling.

### Food restriction

At 7 weeks of age, mice were transitioned from ad libitum food access to restricted chow access. Mice were fed standard chow that weighed 20% of the average mouse body weight (Approximately 4 g chow for males and 3.5 g chow for females per day) following daily VOMA sessions. This feeding schedule controls appetite without hindering weight gain throughout the VOMA paradigm [45]. Mice were fed 1–2 g of mouse chow per day until they reached and maintained 85% of their baseline body weight and then began radial arm maze (RAM) assessments. Baseline working memory assessments on the 8-arm radial maze took place over 4d Mice resumed daily feeding of chow weighing 20% of their body weight following all RAM assessments.

### Radial 8-arm maze shaping

The RAM was used to assess spatial working memory, as described previously [45], using Maypo instant oatmeal cereal (Homestat Farm, Dublin, OH) as food rewards. Prior to the start of RAM assessments, mice were food restricted for 3 days until they reached 85% of their baseline body weight. Chow intake during this pre-RAM period was controlled by feeding mice 10% of their BW (~1.5 to ~2.5 gs per mouse), at least 16 h prior to the start of RAM. On day 1 of RAM shaping, mice were placed on the RAM for 5 min (each) to acclimate to the maze and room cues, with food baits available all around the maze and inside food cups. On day 2 of shaping, mice were given 15 min each to explore the maze and obtain the food baits located only at ends of maze-arms and inside cups. Baseline RAM performance was scored on days 3–4, when mice were given 15 min to collect food rewards found only inside submerged cups at the ends of all 8 arms.

### Groups

Mice were assigned to drug and control groups, balanced across treatments using body weights and baseline working memory performance. VOMA groups contained *n* = 14 mice per sex and Control groups (no meth, Ensure only (Abbot Laboratories)) contained *n* = 6 and *n* = 5 mice, for males and females respectively.

### Methamphetamine formulation and voluntary oral administration protocol

A stock solution of 40 mg/mL of methamphetamine hydrochloride (Sigma Aldrich) was formulated using de-ionized water. Working-MA solutions were diluted into vanilla-flavored Ensure (Abbot Laboratories), using daily average body weights to calculate MA concentrations daily. Ensure-MA solutions were prepared containing 0.6 to 4 mg/mL of MA, to administer 0.25 to 1 mg/kg of MA in a 7*μ*L volume per MA presentation (MA bait), which we have previously identified as optimal for this voluntary oral administration method. MA presentations were delivered to mice in polystyrene petri dishes with petri dishes being cleaned with sterile water and sterile cleaning wipes at the end of every MA presentation. *Escalation phase of VOMA:* mice received 1 dose/day of 0.25 mg/kg MA per bait for 3 days followed by 4 doses/day of 0.25 mg/kg MA per bait for 3 days. Next, mice received 16 doses/day of 0.25 mg/kg MA per bait for 2 days and 16 doses/day of 0.5 mg/kg MA per bait for the last 2 days of the escalation phase of VOMA*. From day 11 to day 28 of VOMA*: mice received 16 doses/day of 1 mg/kg MA per bait, comprising the static phase of VOMA. During VOMA, MA presentations were delivered during a 4 h time window. For experimental days involving 16 MA presentations, baits were delivered at 15 min intervals and at the end of every 15 min interval, visual inspection of petri dishes allowed for tracking of voluntary consumption of the MA baits. For each MA presentation that was delivered, consumption was recorded and tracked throughout the 28d administration period. Control mice had vehicle (Ensure presentations only; 7*μ*L/bait) delivered at the same rate and described procedure as MA treated mice.

### RAM working memory assessment

Working memory assessments occurred 14d after completing VOMA as in our previous studies [25,45]. Mice were tested 2 times (1 trial/day). Each trial started with all food cups baited with Maypo instant oatmeal cereal. Mice were held for 30 s in the center platform with a plastic cylinder, before allowed to retrieve the food rewards from all 8 arms. Mice were only allowed to collect baits from up to three sequential arms before the experimenter interrupted the chaining strategy, to prevent a non-spatial chaining strategy. Working memory errors were recorded as re-entries into arms where the food reward had already been collected during the test trial. The maximum latency for each trial was set to 15 min.

### Tissue sample collection and fractionation

Following 28d of VOMA and 15 days of abstinence, all mice were euthanized and hippocampi and nucleus accumbens from all animals were collected. Whole hippocampi and nucleus accumbens were flash frozen on dry ice and stored frozen at −80 °C, until fractionated into cytosolic and synaptic samples as previously reported [25,45]. Hippocampal and accumbal tissue were immersed in homogenization buffer (Tris 50 mM; EDTA 1 mM; EGTA 1 mM), composed of a SigmaFast protease inhibitor cocktail (Sigma Aldrich) which also contained AEBSF (2 mM), Phosphoramidon (1 mM), Bestatin (130 mM), E-64 (14 mM), Leupeptin (1 mM), Aprotinin (0.2 mM), and Pepstatin A (10 mM). Homogenization was completed using 200 μl of TEE-homogenization buffer and a motorized pestle. Homogenates were transferred to Eppendorf tubes and centrifuged at 3000 g for 5 min at 4 °C. The resulting supernatant underwent a secondary centrifugation at 100,000 g for 30 min at 4 °C. From this second centrifugation step, the supernatant was collected, labeled, and stored as the cytosolic fraction. The pellet that remained was resuspended in 100 μl TEE buffer that also contained 0.001% Triton X-100 and incubated on ice for 1 h before continuing the procedure. This resuspended pellet was then centrifuged once more at 100,000 g for 1 h at 4 °C. The resulting pellet from this third centrifugation step was suspended in 50 μl of TEE buffer, labeled, and stored as the synaptic fraction [25,45]. Following tissue fractionation, the bicinchoninic acid assay (BCA, Thermo Scientific, Rockford, IL) was used to determine protein concentration for each cytosolic and synaptic sample, in order to prepare equivalent loading preparations for western blotting. Samples were finally reduced with 4x Laemmli sample buffer equivalent to 25% of the total volume of the sample, boiled, and stored frozen at −80 °C until used for western blotting.

### Protein quantification and western blot assessments

Tris/Glycine 4–20% gels (Invitrogen) were used to probe synaptic and cytosolic samples of hippocampal homogenates containing 20 μg of protein (each). We resolved GAPDH (36 kDa), *α*-Tubulin (55 kDa), GluA1 (100 kDa), GluA2 (102 kDa), GSK3*β* (46 kDa), and phosphorylated s9 GSK3*β* (46 kDa). Every gel contained 3–4 lanes loaded with the same control sample, (all brain samples, ABS). ABS were used to standardize protein density signals between gels. Proteins on gels were transferred to nitrocellulose membranes using the IBlot^®^ Dry Blotting System (Life Technologies; Carlsbad, CA) using the 9-minute preset program. Nitrocellulose membranes were then incubated in a solution containing 5% sucrose, Tris Buffered Saline with Tween-20 (TBST; 0.1% Tween-20 in TBS) for 30 min at room temperature on a rocker. Samples were incubated with the following primary antibodies for 18–36 hrs at 4 °C: GAPDH (1:2000; Abcam Inc., Cambridge, MA, USA), *α*-Tubulin (1:2000; Calbiochem, San Diego, CA, USA), GluA1 (1:2000; Abcam Inc., Cambridge, MA, USA), *α*-Tubulin (1:2000; Calbiochem, San Diego, CA, USA), GluA2 (1:2000; Chemicon, Temecula, CA, USA), GSK3*β* (1:2000; Cell Signaling Technology, Danvers, MA, USA), and phosphorylated s9- GSK3*β* (1:2000; Cell Signaling Technology, Danvers, MA, USA). Then, membranes were washed in TBST for 20 min and probed with Horseradish Peroxidase (HRP) conjugated secondary antibody. Membranes were incubated with Enhanced Chemiluminescence (ECL) substrate and exposed on CL-XPosure Film (Thermo Scientific; Rockford, IL). GAPDH was used as a control to standardize for protein concentration loaded on gels. Films were analyzed for protein densitometry with ImageJ (NIH).

### Statistical analyses

For MA consumption and behavioral assessments, ANOVAs were used to analyze differences between-subjects and within-subjects data using GraphPad’s Prism Ver. 8 (La Jolla, California). Correlations between MA consumption and working memory errors used Pearson correlations with 95% CIs. Western blot data was analyzed using between-subjects ANOVA analyses. Post-hoc comparisons used Bonferroni-corrected t-tests. Where noted, control data was used to normalize VOMA behavior and western blot data to a% of the average control.

## Results

### Experimental timeline and body weights

**Fig. 1.**
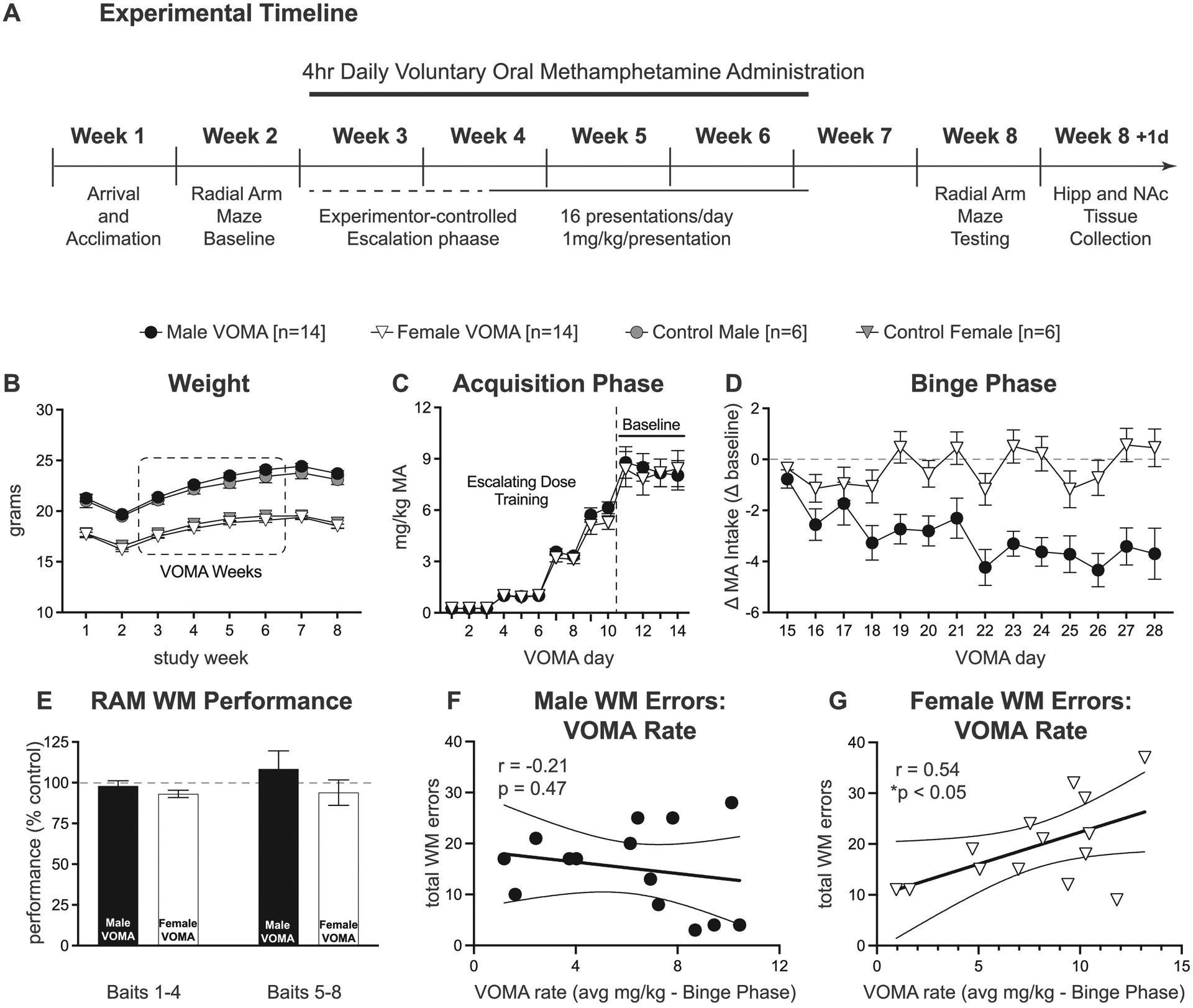
The escalation voluntary oral methamphetamine administration (VOMA) model produces increased consumption in female VOMA mice accompanied by increased total WM errors on the RAM. The paradigm lasted 8 weeks, with acclimation and shaping during weeks 1–2; VOMA during weeks 3–6; behavioral assessments during weeks 7–8 (a). Body weights were tracked across all eight weeks, and there were no significant differences between drug and control mice (b). The acquisition phase of VOMA was characterized from days 1–14 with the average MA consumption over days 11–14 designated as baseline consumption per animal. There was a no significant effect of sex (male vs female; F_1,26_=0.20, *p* = 0.66), no significant sex by day interaction (F_13,338_=0.32, *p* = 0.99) but a significant effect of day (days 1–14; F_13,338_=125.20, *p*<0.0001) during the acquisition phase of VOMA with no significant post-hoc differences (c). The binge phase of VOMA was characterized from days 15–28. Difference scores of MA intake per day from baseline consumption per animal (Δ consumption) were calculated for each day from days 15–28. There was a significant effect of sex (male vs female; F_1,26_=14.70, *p*<0.001), a significant effect of day (days 15–28; F_13,338_=4.07, *p*<0.001), and a significant interaction effect of sex by day (F_13,338_=3.78, *p*<0.001) during the binge phase of VOMA. Females consumed significantly more MA compared to baseline consumption compared to males on days 18, 21–24, and 26–28, *p*<0.05 (d). Working-memory performance on the RAM was assessed across baits 1–4 and baits 5–8. WM performance is shown as a percent of sex-specific control groups. There was no significant effect of bait (F_1,26_=0.66, *p* = 0.42), no effect of sex (F_1,26_=1.79, *p* = 0.19), and no bait by sex interaction (F_1,26_=0.48, *p* = 0.49) (e). Analyses of total number of working memory errors on the RAM correlated to average MA consumption during the binge phase for male VOMA mice revealed no significant correlation between total WM errors and binge consumption rate (r_12_=−0.21, *p* = 0.47) (f). Analyses of total number of working memory errors on the RAM correlated to average MA consumption during the binge phase for female VOMA mice revealed a significant correlation between total WM errors and binge consumption rate (r_12_=0.54, *p*<0.05, *) (g).

The timeline depicts our paradigm and the progression through each in-vivo assessment over 57d [Fig 1A]. Fig. 1B shows analyses of daily body weights for male VOMA mice and control mice across 8 weeks of the study. A two-way ANOVA for male mice revealed no significant effect of drug (MA vs control; F_1,18_=0.83, *p* = 0.37) nor a significant interaction effect of drug by week (F_7,126_=0.99, *p* = 0.44) but a significant effect of week (weeks 1–8; F_2.56,46.71_=212.40, *p*<0.0001) with no significant post-hoc differences. A two-way ANOVA for female mice revealed no significant effect of drug (MA vs control; F_1,18_=0.86, *p* = 0.37) nor a significant interaction effect of drug by week (F_7,126_=0.29, *p* = 0.97) but a significant effect of week (weeks 1–8; F_2.05,36.94_=77.14, *p*<0.0001) with no significant post-hoc differences. Results indicate that food restriction for all treatment groups throughout the paradigm (fed 20% of BW, daily) as well as for RAM assessments (fed 10% of BW, 3d until reached 85% baseline BW) did not result in significant differences in BW between VOMA and control mice at any timepoint throughout the duration of the eight-week study.

### VOMA consumption acquisition and binge phases

A total of 28 mice began and completed the VOMA paradigm (*n* = 14 males, *n* = 14 females). Results indicate that male VOMA mice consumed at variable rates ranging from 1.5 mg/kg MA/day to 7.6 mg/kg MA/day and an average rate of 4.7 mg/kg MA/day over 28d Female VOMA mice consumed at variable rates ranging from 1.1 mg/kg MA/day to 9.6 mg/kg MA/day and an average rate of 5.8 mg/kg MA/day over 28d The first 10d of the VOMA paradigm were considered the acquisition phase where escalation dose training occurred for all mice to acclimate to the paradigm. The average consumption from days 11–14 was considered the baseline consumption rate per animal to confirm that all mice acquired the VOMA task before entering the static dosing schedule. Days 15–28 were considered the binge phase where static dosing occurred for the remainder of the paradigm. The difference score of MA intake per day from baseline consumption per animal (Δ consumption) was calculated for days 15–28. A two-way ANOVA of consumption during the acquisition phase revealed no significant effect of sex (male vs female; F_1,26_=0.20, *p* = 0.66), no significant sex by day interaction (F_13,338_=0.32, *p* = 0.99) but a significant effect of day (days 1–14; F_13,338_=125.20, *p*<0.0001) with no significant post-hoc comparisons [Fig 1C]. A two-way ANOVA of the Δ consumption during the binge phase revealed a significant effect of sex (male vs female; F_1,26_=14.70, *p*<0.001), a significant effect of day (days 15–28; F_13,338_=4.07, *p*<0.001), and a significant sex by day interaction (F_13,338_=3.78, *p*<0.001) [Fig 1D]. Post-hoc analyses revealed females consumed significantly more MA, represented as a Δ from baseline MA consumption, compared to males on days 18, 21–24, and 26–28, *p*<0.05.

### RAM working memory assessment

Working-memory performance on the RAM following two weeks of abstinence from VOMA was analyzed across baits, i.e. arms visited to collect the first four baits (baits 1–4) and the second four baits (baits 5–8). Within each bait set sequence, the number of correct arms (*n* = 4) was divided by the total number of arms visited to collect the first four bait pairs and last four bait pairs. Previous results on the RAM following VOMA demonstrated that mice had a higher performance after collecting the first four baits, when the working memory load was low, compared to the last four baits when the working memory load was high [45]. WM performance is shown as a percent of sex-specific control groups. A two-way repeated measures ANOVA of WM performance between sex and baits [Fig 1E] reveals no significant effect of bait (F_1,26_=0.66, *p* = 0.42), no effect of sex (F_1,26_=1.79, *p* = 0.19), and no interaction effect of bait by sex (F_1,26_=0.48, *p* = 0.49). Analyses of total number of working memory errors on the RAM correlated with average MA consumption during the binge phase for male VOMA mice [Fig 1F] revealed no significant correlation between total WM errors and binge consumption rate (r_12_=−0.21, *p* = 0.47). Analyses of total number of working memory errors on the RAM correlated with average MA consumption during the binge phase for female VOMA mice [Fig 1G] revealed a significant correlation between total WM errors and binge consumption rate (r_12_=0.54, *p*<0.05, *). These results highlight that during the binge phase of Escalation VOMA, female mice consume more MA than males which is associated with increased working memory deficits, highlighting female susceptibility to the cognitive dysfunction following abstinence from chronic VOMA.

### Hippocampal and accumbal GluA1 and GluA2 expression

**Fig. 2.**
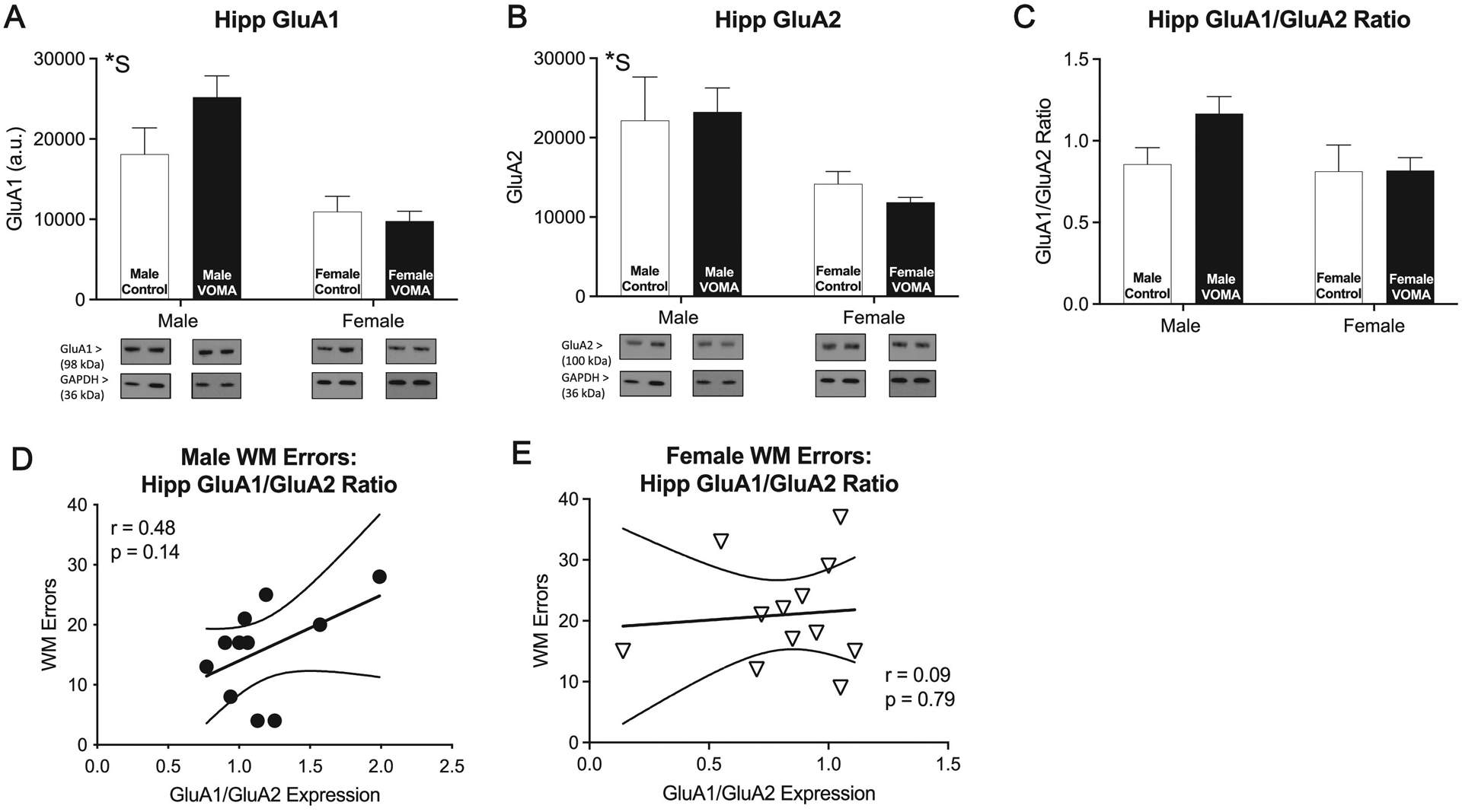
AMPAR signaling in the hippocampus demonstrates baseline sex differences. Hippocampal GluA1 (a) and GluA2 expression (b) is significantly lower in female mice compared to male mice. There was no difference in the hippocampal ratio of GluA1/GluA2 (c). There was no significant correlation between total working memory errors and the ratio of hippocampal GluA1/GluA2 expression in males (r_9_=0.48, *p* = 0.14), (d) nor in females (r_10_=0.09, *p* = 0.79) (e). VOMA: voluntary oral methamphetamine administration. * *S*=overall effect of sex, * *D*=overall effect of drug, *S x * *D*= interaction effect of sex by drug. ** *p*<0.01, *** *p <* 0.001.

Hippocampal synaptic fractions were analyzed for expression of to investigate sex differences in molecular mechanisms underlying VOMA induced working memory deficits. GluA1 and GluA2 are markers known to be involved in synaptic plasticity for short-term and long-term memory respectively [40,41]. A two-way repeated measures ANOVA of hippocampal GluA1 expression [Fig. 2A] reveals no significant effect of drug (F_1,26_=1.13, *p* = 0.29), a significant effect of sex (F_1,26_=16.25, ***p<0.001), and no interaction effect of drug by sex (F_1,26_=2.12, *p* = 0.15). A two-way repeated measures ANOVA of hippocampal GluA2 expression [Fig. 2B] reveals no significant effect of drug (F_1,26_=0.04, *p* = 0.84), a significant effect of sex (F_1,26_=10.16, **p<0.01), and no interaction effect of drug by sex (F_1,26_=0.32, *p* = 0.58). A two-way repeated measures ANOVA of the ratio of hippocampal GluA1/GluA2 expression [Fig. 2C] reveals no significant effect of drug (F_1,26_=1.45, *p* = 0.23), no significant effect of sex (F_1,26_=2.24, *p* = 0.15), and no interaction effect of drug by sex (F_1,26_=1.35, *p* = 0.26). Female mice display lower GluA1 and GluA2 expression in the hippocampus compared to males. Analyses of total number of working memory errors on the RAM correlated with the ratio of hippocampal GluA1/GluA2 expression [Fig. 2D] revealed no significant correlation in males (r_9_=0.48, *p* = 0.14), nor in females (r_10_=0.09, *p* = 0.79) [Fig. 2E].

To control for baseline sex differences in AMPAR signaling, west-ern blot expression for VOMA mice was normalized to sex specific controls and analyzed as a% of control to further investigate AMPAR expression following abstinence from VOMA. A one-tailed *t*-test between hippocampal GluA1 expression [Supplemental Fig S1A] in VOMA males and females reveals a significant difference between sexes (t_21_=2.73, **p<0.01). Female VOMA mice had significantly lower hippocampal GluA1 expression compared to VOMA males. A one-tailed *t* test between hippocampal GluA2 expression [Supplemental Fig S1B] in VOMA males and females reveals no significant difference between sexes (t_24_=1.55, *p>*0.05). A one-tailed *t*-test between the ratio of hippocampal GluA1/GluA2 expression [Supplemental Fig S1E] in VOMA males and females reveals a significant difference between sexes (t_21_=2.28, *p<0.05). Female VOMA mice had a significantly lower ratio of hippocampal GluA1/GluA2 expression compared to VOMA males. Taken together these results suggest sex differences in basal levels of hippocampal GluA1 and GluA2 expression as well as a sex specific decrease in AMPAR expression in the hippocampus following withdrawal from chronic MA.

**Fig. 3.**
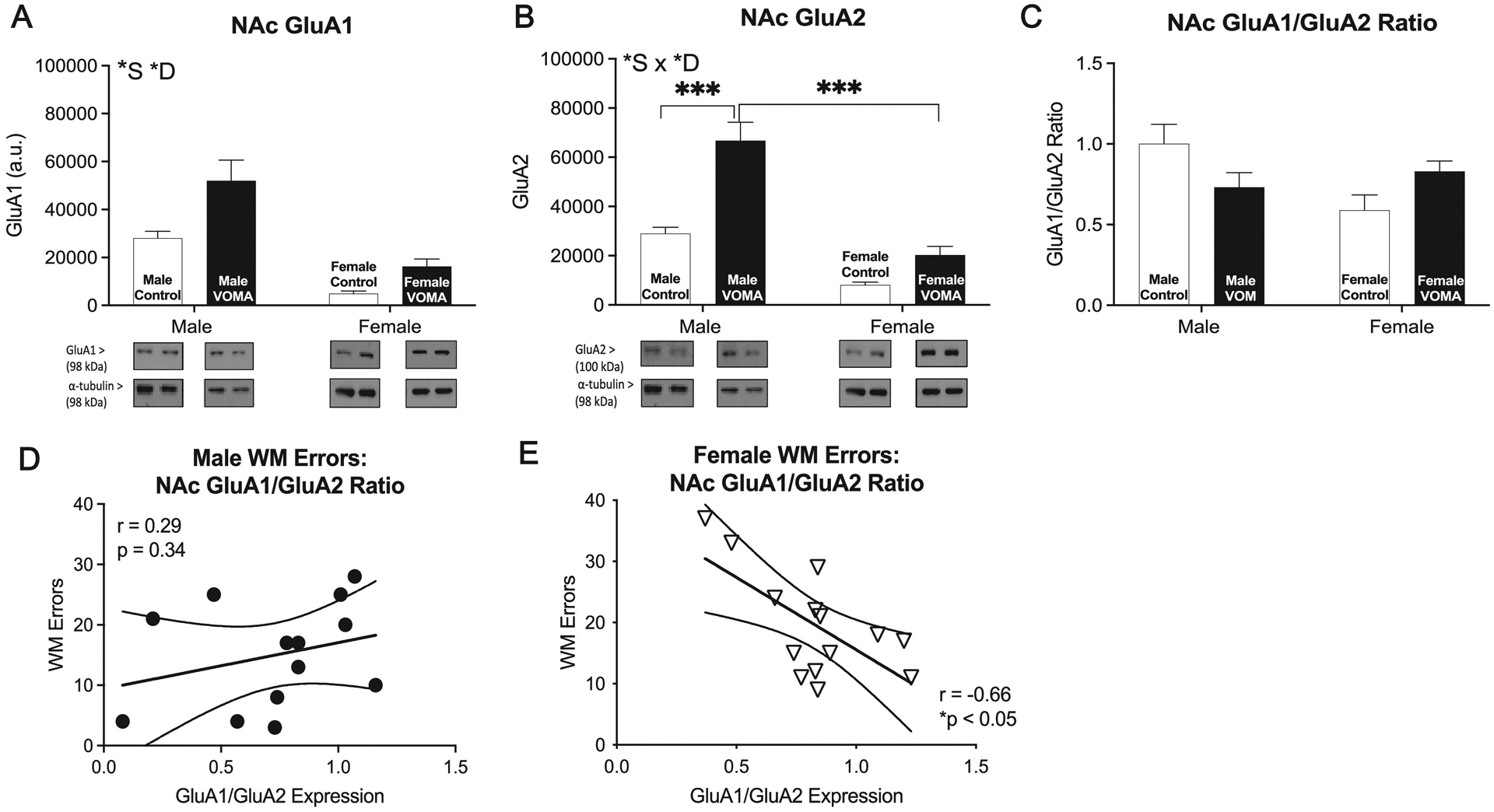
Abstinence from VOMA produces female specific decreases in GluA2 signaling in the nucleus accumbens compared to males and a correlation to working memory errors. Female mice demonstrate decreased GluA1 expression (a) compared to males with abstinence from VOMA producing overall increases in GluA1 expression in the nucleus accumbens. Female VOMA mice display decreased GluA2 (b) expression in the nucleus accumbens compared to male VOMA mice with no differences in the ratio of GluA1/GluA2 (c). There was no significant correlation between total working memory errors and the ratio of accumbal GluA1/GluA2 expression in males (r_11_=0.29, *p* = 0.34) (d), however, there was a significant correlation found in females (r_11_=−0.66, * *p*<0.05) (e). Female VOMA mice displayed more working memory errors correlated with a lower accumbal GluA1/GluA2 ratio. VOMA: voluntary oral methamphetamine administration. * *S*=overall effect of sex, * *D*=overall effect of drug, *S x * *D*= interaction effect of sex by drug. ** *p*<0.01, *** *p <* 0.001.

NAc synaptic fractions were analyzed for expression of GluA1 and GluA2 to investigate sex differences in molecular mechanisms underlying VOMA induced changes in reward circuitry and drug seeking. GluA1 and GluA2 are markers known to be involved in promoting drug craving and the incubation of craving of MA [50,55]. A two-way repeated measures ANOVA of accumbal GluA1 expression [Fig. 3A] reveals a significant effect of drug (F_1,35_=6.56, **p<0.01), a significant effect of sex (F_1,35_=18.36, ***p<0.0001), and no interaction effect of drug by sex (F_1,35_=0.84, *p* = 0.37). A two-way repeated measures ANOVA of accumbal GluA2 expression [Fig. 3B] reveals a significant effect of drug (F_1,35_=16.16, ***p<0.001), a significant effect of sex (F_1,35_=29.56, ***p<0.0001), and an interaction effect of drug by sex (F_1,35_=4.28, *p*<0.05, *). Post-hoc analyses revealed that female VOMA mice had significantly lower GluA2 expression compared to VOMA males (t_35_=6.76, ***p<0.0001). A two-way repeated measures ANOVA of the ratio of accumbal GluA1/GluA2 expression [Fig. 3C] reveals no significant effect of drug (F_1,35_=0.02, *p* = 0.87), no significant effect of sex (F_1,35_=2,62, *p* = 0.11), and an interaction effect of drug by sex (F_1,35_=6.96, *p<0.05), with no post-hoc differences. Similar to what was observed in the hippocampus, female mice have less GluA1 and GluA2 expression in the NAc compared to males. Additionally, VOMA mice exhibit overall increases in AMPAR signaling in the nuceleus accumbens compared to controls following abstinence from VOMA. Analyses of total number of working memory errors on the RAM correlated with the ratio of accumbal GluA1/GluA2 expression [Fig. 3D] revealed no significant correlation in males (r_11_=0.29, *p* = 0.34), however, there was a significant correlation found in females (r_11_=−0.66, *p<0.05). More working memory errors were correlated with a lower accumbal GluA1/GluA2 ratioin female VOMA mice following abstinence, indicating lower levels of GluA1 in the nucleus accubens compared to GluA2 expression levels [Fig. 3E].

To control for baseline sex differences in AMPAR signaling, western blot expression for VOMA mice was normalized to sex specific controls and analyzed as a% of control to further investigate AMPAR expression following abstinence from VOMA. A one-tailed *t*-test between accumbal GluA1 expression [Supplemental Fig S1C] in VOMA males and females reveals a significant difference between sexes (t_25_=2.00, *p<0.05). Female VOMA mice had significantly higher accumbal GluA1 expression compared to VOMA males. A one-tailed *t*-test between accumbal GluA2 expression [Supplemental Fig S1D] in VOMA males and females reveals no significant difference between sexes (t_25_=0.93, *p>*0.05). A one-tailed *t*-test between the ratio of accumbal GluA1/GluA2 expression [Supplemental Fig S1F] in VOMA males and females reveals a significant difference between sexes (t_25_=4.77, ***p<0.001). Female VOMA mice had a significantly higher ratio of accumbal GluA1/GluA2 expression compared to VOMA males. Results indicate a sexual disparity in basal levels of AMPAR in the NAc and that VOMA emhances AMPAR signaling in both sexes following abstinence from chronic VOMA. When baseline differences are controlled for, female mice exhibit enhanced AMPAR signaling in the NAc compared to males following withdrawal from VOMA.

### Hippocampal and accumbal GSK3β and p-GSK3β expression

**Fig. 4.**
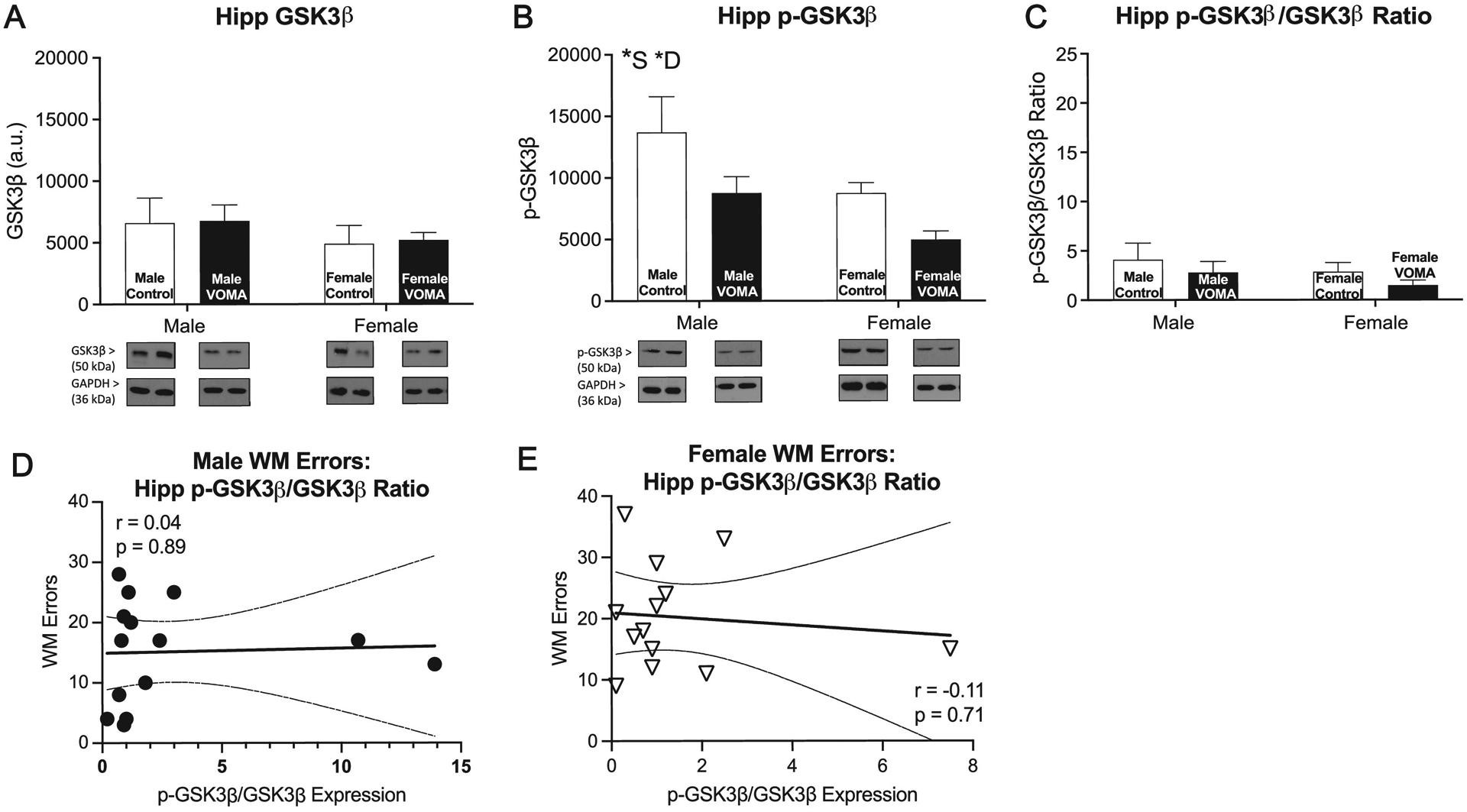
p-GSK3*β* signaling is dysregulated following abstinence from chronic VOMA. Female mice are more vulnerable to Hippocampal GSK3*β* expression (a) does not significantly change whereas mice display decreased p-GSK3*β* expression (b) with female VOMA mice displaying less p-GSK3*β* compared to male mice following abstinence and no differences in the ratio of p-GSK3*β*/GSK3*β* expression. There was no significant correlation between total working memory errors and the ratio of hippocampal p-GSK3*β*/GSK3*β* expression in males (r_12_=0.04, *p* = 0.89), nor in females (r_11_=−0.11, *p* = 0.71). VOMA: voluntary oral methamphetamine administration. * *S*=overall effect of sex, * *D*= overall effect of drug, *S x * *D*= interaction effect of sex by drug. ** *p*<0.01, *** *p <* 0.001.

Hippocampal synaptic fractions were analyzed for expression of GSK3*β* and p-GSK3*β*, markers known to be involved in regulation of synaptic plasticity and AMPAR membrane destabilization [44,57]. A two-way repeated measures ANOVA of hippocampal GSK3*β* expression [Fig 4A] reveals no significant effect of drug (F_1,36_=0.29, *p* = 0.87), no significant effect of sex (F_1,36_=1.48, *p* = 0.23), and no interaction effect of drug by sex (F_1,36_=0.002, *p* = 0.96). A two-way repeated measures ANOVA of hippocampal p-GSK3*β* expression [Fig. 4B] reveals a significant effect of drug (F_1,36_=8.59, *p*<0.01, **), a significant effect of sex (F_1,36_=8.65, *p*<0.01, **), and no interaction effect of drug by sex (F_1,36_=0.15, *p* = 0.70). Female mice had less hippocampal p-GSK3*β* expression compared to males. VOMA mice displayed decreased p-GSK3*β* compared to controls. A two-way repeated measures ANOVA of the ratio of hippocampal p-GSK3*β*/GSK3*β* expression [Fig. 4C] reveals no significant effect of drug (F_1,36_=1.46, *p* = 0.24), no significant effect of sex (F_1,36_=1.33, *p* = 0.26), and no interaction effect of drug by sex (F_1,36_=0.001, *p* = 0.97). Analyses of total number of working memory errors on the RAM correlated with the ratio of hippocampal p-GSK3*β*/GSK3*β* expression [Fig. 4D] revealed no significant correlation in males (r_12_=0.04, *p* = 0.89), nor in females (r_11_=−0.11, *p* = 0.71) [Fig. 4E].

To control for baseline sex differences in GSK3*β* signaling, western blot expression for VOMA mice was normalized to sex specific controls and analyzed as a% of control to further investigate GSK3*β* signaling following abstinence from VOMA. A one-tailed *t*-test between hippocampal GSK3*β* expression [Supplemental Fig S2A] in VOMA males and females reveals no significant difference between sexes (t_26_=0.16, *p>*0.05). A one-tailed *t*-test between hippocampal p-GSK3*β* expression [Supplemental Fig S2B] in VOMA males and females reveals no significant difference between sexes (t_26_=0.58, *p>*0.05). A one-tailed *t*-test between the ratio of hippocampal p-GSK3*β*/GSK3*β* expression [Supplemental Fig S2E] in VOMA males and females reveals no significant difference between sexes (t_26_=0.50, *p>*0.05).

**Fig. 5.**
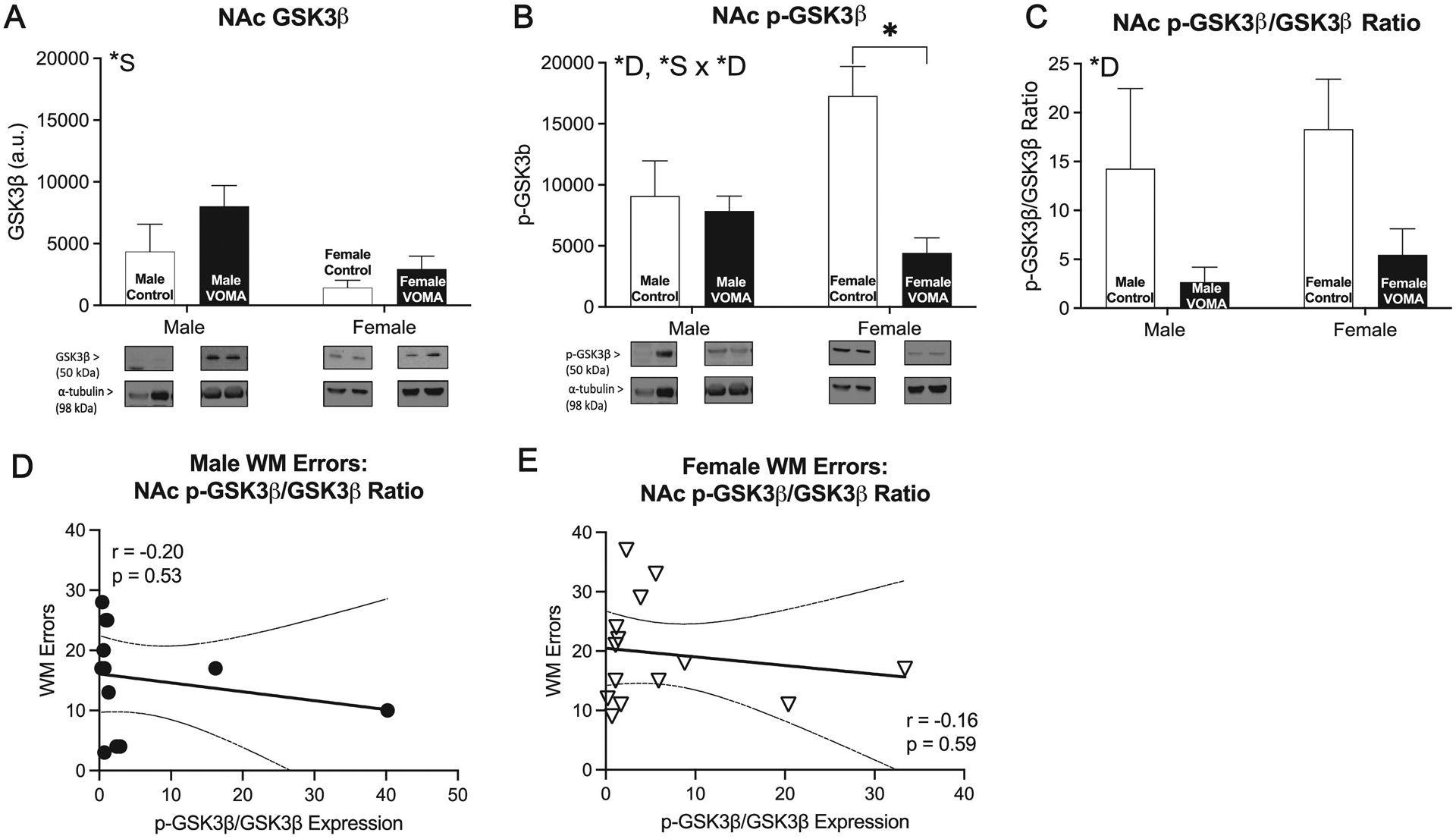
Abstinence from VOMA produces female specific decreases in p-GSK3*β* signaling in the nucleus accumbens. Female mice demonstrate decreased GSK3*β* (*a*) expression in the nucleus accumbens compared to males. Female VOMA mice display decreased p-GSK3*β* (*b*) expression in the nucleus accumbens compared to male mice, with both sexes decreasing the ratio of p-GSK3*β*/GSK3*β* following abstinence (c). There was no significant correlation between total working memory errors and the ratio of accumbal p-GSK3*β*/GSK3*β* expression in males (r_10_=−0.20, *p* = 0.53), nor in females (r_12_=−0.16, *p* = 0.59). VOMA: voluntary oral methamphetamine administration. * *S*=overall effect of sex, * *D*= overall effect of drug, *S x * *D*= interaction effect of sex by drug. ** *p*<0.01, *** *p <* 0.001.

NAc synaptic fractions were analyzed for expression of GSK3*β* and p-GSK3*β*, markers known to be involved in activation of neurotoxic signaling cascades as well as promoting downstream apoptosis and inflammation [44]. A two-way repeated measures ANOVA of accumbal GSK3*β* expression [Fig. 5A] reveals no significant effect of drug (F_1,32_=2.31, *p* = 0.14), a significant effect of sex (F_1,32_=5.60, *p<0.05), and no in-teraction effect of drug by sex (F_1,32_=0.41, *p* = 0.53). Female mice had lower accumbal GSK3*β* expression compared to males. A two-way repeated measures ANOVA of accumbal p-GSK3*β* expression [Fig. 5B] reveals a significant effect of drug (F_1,32_=14.75, ***p<0.001), no significant effect of sex (F_1,32_=1.69, *p* = 0.20), and a significant interaction effect of drug by sex (F_1,32_=10.08, **p<0.01). VOMA mice had decreased p-GSK3*β* expression compared to controls. A two-way repeated measures ANOVA of the ratio of accumbal p-GSK3*β*/GSK3*β* expression [Fig. 5C] reveals a significant effect of drug (F_1,32_=8.72, **p<0.01), no significant effect of sex (F_1,32_=0.68, *p* = 0.42), and no significant interaction effect of drug by sex (F_1,32_=0.02, *p* = 0.88), with no post-hoc differences. Analyses of total number of working memory errors on the RAM correlated with the ratio of accumbal p-GSK3*β*/GSK3*β* expression [Fig. 5D] revealed no significant correlation in males (r_10_=−0.20, *p* = 0.53), nor in females (r_12_=−0.16, *p* = 0.59) [Fig. 5E].

To control for baseline sex differences in GSK3*β* signaling, western blot expression for VOMA mice was normalized to sex specific controls and analyzed as a% of control to further investigate GSK3*β* signaling following abstinence from VOMA. A one-tailed *t*-test between accumbal GSK3*β* expression [Supplemental Fig S2C] in VOMA males and females reveals no significant difference between sexes (t_24_=0.22, *p>*0.05). A one-tailed *t*-test between accumbal p-GSK3*β* expression [Supplemental Fig S2D] in VOMA males and females reveals a significant difference between sexes (t_24_=2.74, **p<0.01). Female VOMA mice had a significantly lower expression of accumbal p-GSK3*β* compared to VOMA males. A one-tailed *t*-test between the ratio of accumbal p-GSK3*β*/GSK3*β* expression [Supplemental Fig S2F] in VOMA males and females reveals no significant difference between sexes (t_26_=0.50, *p>*0.05). Results suggest that decreased accumbal p-GSK3*β* signaling may produce enhanced female specific cell death, neurotoxicity, and inflammation following abstinence from chronic VOMA.

## Discussion

Our study investigated sex differences in short-term memory performance and the associated molecular signaling cascades regulating synaptic plasticity and an addictive phenotype following two weeks of abstinence from chronic voluntary oral methamphetamine administration (VOMA) in young-adult male and female mice (6–12 weeks of age). Our analyses revealed sex-specific effects on MA consumption associated with more WM errors, and sex-specific shifts in neurochemical markers in the hippocampus and NAc following abstinence from VOMA. Consumption data show that female mice consumed more MA compared to male mice in the binge phase of VOMA, a result that was correlated with increased working memory errors on the RAM spatial short-term memory task. Furthermore, sex differences in basal levels of AMPARs in the hippocampus and NAc suggest female specific vulnerability to an addictive phenotype and potential exacerbation of spatial working memory deficits in females compared to males. Additionally, sex differences in basal levels of p-GSK3*β* and decreased p-GSK3*β* expression in the NAc of female mice following withdrawal from MA may indicate female-specific vulnerability to downstream neurotoxic signaling cascades, such as apoptosis and inflammation, following chronic MA use. These results highlight the female-specific cognitive susceptibility to the chronic effects of VOMA and sex differences in the long-term differential neurochemical modulations involved in memory dysfunction and persistent MA-induced modulation of behavioral phenotypes present during periods of withdrawal.

### 28d of VOMA increases female MA preference in mice but does not induce body weight changes

The present study found that following two weeks of forced abstinence from 28d of VOMA, comprised of an acquisition phase for 14d with escalating doses and a binge phase for 14d with static doses, produced an increase in MA consumption observed in female mice only. Our data reveal significant sex differences in the MA consumed daily during the binge phase of VOMA days 15–28 compared as a daily change from the average baseline consumption over days 11–14 of the VOMA paradigm per animal. In our VOMA study, during the acquisition phase (first 14d of VOMA) female mice consumed on average 3.51 mg/kg MA/day whereas male mice consumed on average 4.00 mg/kg MA/day. Male and female mice in this paradigm did not consume differently during this phase of the task. Although our paradigm did not produce disparities in acquisition of drug abuse as reported in the human literature, all animals did acquire the appetitive task and were able to progress on to the binge phase of the paradigm where significant consumption differences were observed. During the binge phase (last 14d of VOMA), female mice consumed on average 7.90 mg/kg MA/day whereas male mice consumed on average 5.30 mg/kg MA/day. Our VOMA model has identified a female-specific vulnerability to the maintenance of drug abuse that is also observed in clinical reports where women consume more MA compared to men, have increased maintenance of MA, and have higher relapse rates following withdrawal compared to men [6,8–10]. Our results add to the existing body of literature on rodent models of voluntary oral administration and reproduce human female-specific vulnerabilities to the phases of MA addiction.

Both male and female VOMA mice did not lose weight compared to control mice throughout the 28d VOMA paradigm. Bolus doses of 5 mg/kg MA and 10 mg/kg MA have been reported to produce weight loss in male mice [58]. However, mice that self-administer similar escalating doses of MA have demonstrated no effect on weight loss and instead demonstrate steady increases of body weight throughout the experimental procedures [59]. Consumption rates in our VOMA study ranged from 1.5 mg/kg MA/day to 7.6 mg/kg MA/day over 28d in male mice with an average rate of 4.7 mg/kg MA/day. In female VOMA mice, consumption rates ranged from 1.1 mg/kg MA/day to 9.6 mg/kg MA/day over 28d with an average rate of 5.8 mg/kg MA/day. The MA consumption rates in the current study are similar to previously reported escalating doses in self-administration models and allow for steady weight maintenance throughout the eight-week study period. Administration of a higher neurotoxic dose or variable dosing schedule may be needed to produce any significant changes in body weight over the duration of the paradigm.

### Decreased basal levels of hippocampal AMPAR expression in female mice suggests vulnerability to synaptic destabilization associated with persistent working memory deficits

We observed increases in total WM errors on the RAM in female VOMA mice that was correlated with a higher binge consumption rate, an effect not observed in males. Additionally, female mice displayed lower basal levels of hippocampal GluA1 and GluA2 and female VOMA mice displayed decreased GluA1 expression in the hippocampus compared to male VOMA mice when basal sex differences were controlled for. The RAM is an appetitive non-aversive memory task used to assess short term spatial memory that has been previously used to test working memory deficits following MA abuse [25,45,58]. We used the RAM to assess short-term working memory to replicate deficits in short term memory recall tasks observed in human MA users following prolonged periods of withdrawal [35]. Our VOMA model has been previously reported to produce short term spatial working memory and reference memory deficits using the RAM following withdrawal from MA in male [45] and female mice [25]. GluA1 and GluA2 promote LTP and LTD, respectively, and are known to be dysregulated following drug abuse [30,49,50]. Our previous findings demonstrated that spatial short-term working memory deficits were associated with decreased hippocampal GluA2 expression and PKM*ζ* expression in male mice promoting synaptic destabilization and modulations in AMPAR trafficking [45]. We also demonstrated that female specific increases in short term spatial WM errors on the RAM following abstinence from VOMA were associated with decreased PKM*ζ* expression in the hippocampus compared to males [45]. The current study expands on these findings and highlights a role of AMPAR signaling in the hippocampus and NAc that may promote an addictive phenotype thus enhancing female vulnerability to the long-term neurotoxic effects associated with withdrawal from chronic MA use. Enhanced female specific cognitive deficits that persisted following abstinence may have been due to PKM*ζ*-mediated disruptions in AMPAR signaling and deficits in LTP [60].

In the current study, female mice displayed lower basal levels of GluA1 and GluA2 in the hippocampus compared to males. Sexual dimorphism in the basal levels of molecular mechanisms underlying self-administration of MA [21–23] and the rewarding properties of cocaine [24] have been previously reported in rodent models of SUDs and highlight female susceptibility to the detrimental effects of MA. Our results expand upon previous findings and identify female specific susceptibility to AMPAR signaling cascades that may be affected and modulated following withdrawal from chronic MA administration. When sex differences are controlled for, female VOMA mice downregulated GluA1 but not GluA2 expression in the hippocampus compared to male mice following forced abstinence from VOMA. Dysregulated AMPAR trafficking may produce enhanced synaptic destabilization with disruptions in LTP in females following two weeks of abstinence from VOMA. These synaptic changes may underlie persistent short-term working memory deficits and highlights AMPAR signaling cascades as a contributor to sex specific cognitive deficits following MA abuse. Additionally, male mice in the current study upregulated GluA1 in the hippocampus following abstinence from VOMA which may promote synaptic stabilization and LTP processes, resulting in fewer total working memory errors on the RAM. Interestingly, we did not observe the previously reported effect of bait on working memory performance, where fewer working memory errors were made in baits 1–4 versus baits 5–8 [25,45] when assessing RAM performance across bait groups between sexes. Using RAM to assess reference memory with four baited reward arms and four unbaited arms may increase the cognitive load producing a more difficult memory task. This high-load cognitive task may better detect overall deficits in working memory between sexes during RAM performance following chronic MA use. Furthermore, no female specific increase in WM errors in baits 5–8 when working memory load is high may be due to the high variability in MA consumption rates. Nevertheless, our results highlight a female-specific vulnerability to persistent memory deficits correlated to increased MA consumption and are in concurrence with the human literature where women display a plethora of cognitive and behavioral deficits compared to men following chronic MA abuse [3,4]. Our findings indicate that our VOMA model accurately recapitulates the consumption patterns of MA users and may provide insight into downstream MA-induced neurological deficits following abstinence, as observed in humans.

### Female specific decreases in basal GluA1 and GluA2 expression in the nucleus accumbens may be associated with vulnerability towards synaptic remodeling and memory deficits

Female mice in the current study had lower basal levels of GluA1 and GluA2 compared to males in the NAc, suggesting female vulnerability and potential sensitivity to MA induced behavioral changes following withdrawal from chronic VOMA. Additionally, female mice had increased GluA1 but not GluA2 expression in the NAc compared to male mice following two weeks of abstinence from MA administration when basal sex differences were controlled for. We hypothesize that basal differences in AMPAR signaling prior to chronic self-administration of MA underlies female susceptibility to MA addiction as well as enhanced downstream craving and withdrawal effects following abstinence from MA, recapitulating female MA users’ behavioral phenotype during periods of withdrawal. The increase of AMPAR subunit GluA1 following withdrawal from chronic MA use when basal differences are controlled for may promote long-term dysregulation of calcium and glutamate release in MSNs of the NAc with downstream effects on neuronal projections to the hippocampus. AMPAR signaling in the NAc perpetuates drug craving and drug-seeking behaviors following withdrawal from MA and is a key component in drug relapse [30,31,49,50]. Rodent studies demonstrate that male rats have increased GluA1 but not GluA2 accumulation in the NAc as well as increased GluA1 translation associated with increased drug seeking behavior following prolonged withdrawal from self-administration of chronic MA [49,50]. Recently, a study by Funke et al., (2023) identified persistent upregulation of CP-AMPAR expression in MSNs in the NAc of both male and female rats following withdrawal from chronic MA which was associated with the incubation of MA craving and alterations in synaptic plasticity. Our results may highlight female susceptibility to these drug induced behaviors following abstinence as well as heightened sensitivity to the rewarding properties of MA due to sexual disparities in basal expression of signaling cascades involved in regulating behavioral phenotypes during periods of withdrawal, however, additional studies are needed to confirm this hypothesis.

Previously, we demonstrated female specific increased locomotor activity on the elevated plus maze following withdrawal from VOMA associated with increased hippocampal *κ*OR expression compared to male mice [25]. We hypothesized that enhanced *κ*OR signaling in the hippocampi of female mice may reflect alterations in NAc dependent circuitry underlying the dysphoric effects of MA associated with withdrawal and compulsive drug seeking behaviors [25]. Recent studies have uncovered female specific enhanced basal expression of prodynorphin and *κ*OR in the hippocampus and prefrontal cortex (PFC) as well as higher basal levels of corticotropin-releasing hormone receptors which were correlated with the incubation of MA craving [21,22]. Results from the current study expand upon our previous findings and add to the current literature in identifying additional molecular mechanisms during abstinence from MA that may be involved in the modulation of drug seeking behavior. Characterizing drug seeking behavior across the phases of addiction and following withdrawal from chronic MA using the VOMA paradigm along with assessment of associated neurochemical modulations in AMPAR signaling and calcium release will aid in elucidating the development of behavioral phenotypes associated with female vulnerability to MA addiction and drug relapse reported in clinical studies. Furthermore, the NAc is known to receive glutamatergic projections from the hippocampus and PFC [30–32], thus future studies that characterize the expression of GluA1 and GluA2 in the PFC following forced abstinence from VOMA will aid in understanding how chronic MA use changes neurochemical signaling across multiple brain regions during drug craving and drug-reinstatement.

Our study also identified the NAc as a vulnerable brain region implicated in female susceptibility to enhanced MA-induced synaptic remodeling associated with more WM deficits on the RAM, an association not observed in the hippocampus or in male mice. Following abstinence from VOMA there was a female specific association between total WM errors and the accumbal AMPAR expression ratio such that more errors were associated with less GluA1 compared to GluA2 expression in the NAc, highlighting the importance of GluA1 in working memory. Lesions of the NAc as well as disruptions of AMPAR transmission in the NAc have been implicated in spatial memory impairments and spatial memory consolidation [28,61,62]. Rats with NMDA-induced excitotoxic lesions of the NAc displayed more WM errors on the RAM [28] as well as behavioral impairments on conditioned freezing tasks compared to controls [28,61]. Furthermore, administration of AMPAR and NMDAR competitive antagonists, 6,7-dinitroquinoxaline-2,3-dione (DNQX) and AP-5 respectively, in the NAc of mice impairs spatial performance and memory consolidation on the morris water maze [62], highlighting the importance of AMPAR signaling and integrity in synaptic plasticity in this region to spatial memory tasks. We hypothesize that female VOMA mice in our study are more susceptible to exacerbated memory deficits due to a higher MA consumption rate that promoted neurotoxicity and AMPAR-dependent synaptic remodeling in the NAc during the chronic administration period resulting in mitigated synaptic plasticity and downstream behavioral impairments. Our results are in accordance with previous literature demonstrating the importance of NAc signaling modulations in MA induced cognitive deficits [63–65] and expand the current literature by identifying novel sex differences in NAc-mediated cognitive deficits following a period of abstinence from chronic MA. Studies that investigate modulations in spine density and AMPAR transmission in MSNs of the NAc following abstinence from chronic VOMA will aid in further understanding of female specific vulnerability to NAc driven behavioral impairments following abstinence from chronic MA use.

### GSK3β signaling in the hippocampus may indirectly regulate synaptic plasticity thus promoting working memory deficits

The present study shows that more total working memory errors over the RAM test period following abstinence was correlated with more MA consumption in VOMA females but not in males. In contrast to our hypothesis, there were no modulations in hippocampal GSK3*β* signaling following withdrawal from VOMA suggesting that synaptic plasticity was not directly modulated by GSK3*β*. These results indicate that GSK3*β* may indirectly modulate synaptic plasticity through shared signaling cascades that are upstream of GSK3*β* known to regulate AMPAR activity. When active, GSK3*β* signaling regulates cell survival as well as synaptic destabilization [57]. Active GSK3*β* promotes synaptic destabilization by activating PICK1-GluA2 binding and endocytosis whereas inactive p-GSK3*β* promotes synaptic stabilization through GRIP1 binding complexes [57]. Also, phosphorylation of GSK3*β* by upstream Akt inactivates GSK3*β* promoting increased synaptic stability [57]. GSK3*β* is known to regulate glutamate release, with active GSK3*β* promoting LTD through decreased glutamate release and inactive GSK3*β* promoting LTP [66]. Coimmunoprecipitation studies between PICK1, GRIP1, and AMPAR subunits GluA1 and GluA2 following withdrawal from 28d of VOMA will aid in determining the molecular mechanism modulating synaptic plasticity. Our results suggest that synaptic plasticity may not be directly regulated through GSK3*β* mediated modulations by mechanisms such as PICK1 and GRIP1 but may be regulated by upstream molecular markers through PI3K-Akt signaling cascades such as dopamine receptors (DR.), NMDA receptors (NMDAr), as well as estrogen receptors (ER) which regulate AMPAR activity.

Dopaminergic signaling through D2 receptors (D2R) negatively regulates GluA1 expression at the cAMP-dependent protein kinase (PKA) site, Ser845 producing attenuation of GluA1 expression [67]. Administering a D2R antagonist increases GluA1 signaling via phosphorylation of GluA1 at Ser845 through PKA-cAMP-regulated phosphoprotein of 32 kDa (DARPP-32) cascades [67]. Chronic MA users display long term dysregulated D2R signaling following withdrawal from MA use [15]. Furthermore, NMDA receptors are activated through MA-induced increases in glutamate and in turn produce long term overactive release of calcium resulting in downstream cell death with concomitant DNA damage following chronic MA use [50]. MA induced dysregulated calcium release through NMDAr has direct implications for AMPAR trafficking [68] and may promote enhanced synaptic destabilization resulting in more short-term working memory deficits as MA consumption increases in female mice. Estrogen acts as both a hormone binding to ERs and as a nuclear transcription factor that regulates protein synthesis and gene expression across brain regions promoting downstream changes in cell survival, apoptosis, neurotransmitter modulation, neuronal growth and differentiation, neuronal excitability, and synaptogenesis [68–70]. Furthermore, estrogen regulates synaptic protein synthesis that produces AMPAR subunits and administration of estradiol produces increased GluA1 protein expression in the hippocampus of mice with concomitant modulation of synaptic plasticity [71]. Interestingly, long term early MA use in juvenile mice throughout adolescence is documented to produce a plethora of detrimental changes in ovarian and reproductive functioning that are implicated in adulthood ovarian health [72]. Investigation of D2R, NMDAr, and ER expression throughout the course of the VOMA paradigm may provide clearer insight into the neurochemical regulators of AMPAR underlying female specific spatial working memory deficits following withdrawal from MA.

### Enhanced active GSK3β signaling in the nucleus accumbens of female mice increases female susceptibility to neurotoxic, apoptotic, and inflammatory pathway activation following withdrawal from chronic MA

We observed increased active GSK3*β* signaling, indicated by down regulated p-GSK3*β* expression, in the NAc of female mice compared to male mice following 14d of withdrawal from chronic MA. GSK3*β* is a constitutively active kinase that is involved in signaling cascades promoting neurotoxicity, apoptosis, and inflammation [57]. When phosphorylated by upstream PI3k-Akt signaling, GSK3*β* becomes inactive, promoting downstream neuroprotection from cell death and inflammation [57]. MA promotes neurotoxic excitotoxicity through glutamate signaling, neurotoxicity via formation of free radicals and reactive oxygen species, activation of caspase3 and caspase9 apoptotic pathways, and proliferation of reactive microglia responsible for proinflammatory cytokine release promoting inflammatory pathway activation [55,56,72]. Higher levels of MA exacerbate persistent neurotoxicity, apoptosis, and inflammation that perpetuates cognitive deficits and behavioral deficits in human MA users [73]. We hypothesize that female specific dysregulated GSK3*β* signaling promotes exacerbated activation of down stream neurotoxic, apoptotic, and inflammatory signaling cascades in the NAc that is directly associated with increased MA consumption during VOMA compared to males. Uninhibited GSK3*β* signaling via upstream inactivation of PI3K-Akt signaling regulates calcium channels and calcium-dependent vesicle exocytosis at the pre-synapse to produce decreased calcium influx [66]. Furthermore, calcium/calmodulin dependent protein kinase kinase (CaMKK) signaling through brain derived neurotrophic factor (BDNF) activated PI3K-Akt signaling cascades are responsible for a multitude of calcium dependent neuronal processes, cell survival processes, and synaptic plasticity processes and is hypothesized to be a master regulator of plasticity processes [74–76]. Downstream BDNF signaling promotes active Akt signaling and concomitant inactivation of GSK3*β* to promote neuronal survival [66]. Future studies investigating PI3K and Akt signaling upstream of GSK3*β* as well as BDNF and CaMKK expression in the NAc following prolonged abstinence from VOMA may help identify molecular targets that exacerbate long term calcium dysfunction that promotes cognitive and behavioral deficits in female MA users following abstinence.

## Conclusions

We used a VOMA model [25,45] to determine sex differences in the long-term behavioral and neurochemical effects following abstinence from chronic voluntary oral MA access and identify sex differences in the resulting neurochemistry of the hippocampus and NAc. VOMA female mice display sustained MA consumption correlated with exacerbated short term spatial working memory deficits on the RAM compared to males. These behavioral disparities may be perpetuated by observed changes and synaptic remodeling of AMPARs across the hippocampus and NAc. Dysregulated GSK3*β* signaling in the NAc of female mice suggests enhanced susceptibility to activation of signaling cascades exacerbating neurotoxicity, apoptosis, and inflammation directly associated with increased MA consumption of female mice. Our results shed light on molecular pathways involved in perpetuation of sex differences in AMPAR signaling and its regulation in synaptic plasticity and behavioral phenotypes during forced abstinence from oral MA consumption. Identifying these neurochemical modulations across sexes in brain regions involved in behavioral impairments in learning, memory, and an addictive phenotype in MA addiction is crucial to developing effective therapeutics for SUDs and the cognitive deficits that accompany them. The current study begins to bridge the gap in understanding and in developing therapeutics beneficial for remediating cognitive deficits and compulsive behaviors across male and female populations. Future work should address the specific neurocircuitry involved in drug seeking behavior across a variety of brain regions critical in substance misuse, including the dorsal striatum, PFC, NAc and hippocampus, to identify specific molecular cascades that perpetuate compulsive behaviors and downstream enhanced neurochemical insults following chronic substance misuse and following a period of withdrawal.

## Supplementary Material

Suppl2

Suppl1

## Data Availability

The raw data supporting the conclusions of this manuscript will be made available upon reasonable request to the corresponding author.
