# Supplementary material for: Synaptic remodeling of GluA1 and GluA2 expression in the nucleus accumbens promotes susceptibility to cognitive deficits concomitant with downstream GSK3β mediated neurotoxicity in female mice during abstinence from voluntary oral methamphetamine
Source: Addict Neurosci. Author manuscript; Available in PMC 2023 Dec 1. (PMC10569060; doi:10.1016/j.addicn.2023.100112)
Supplement: Suppl2 [file NIHMS1925481-supplement-Suppl2.docx]

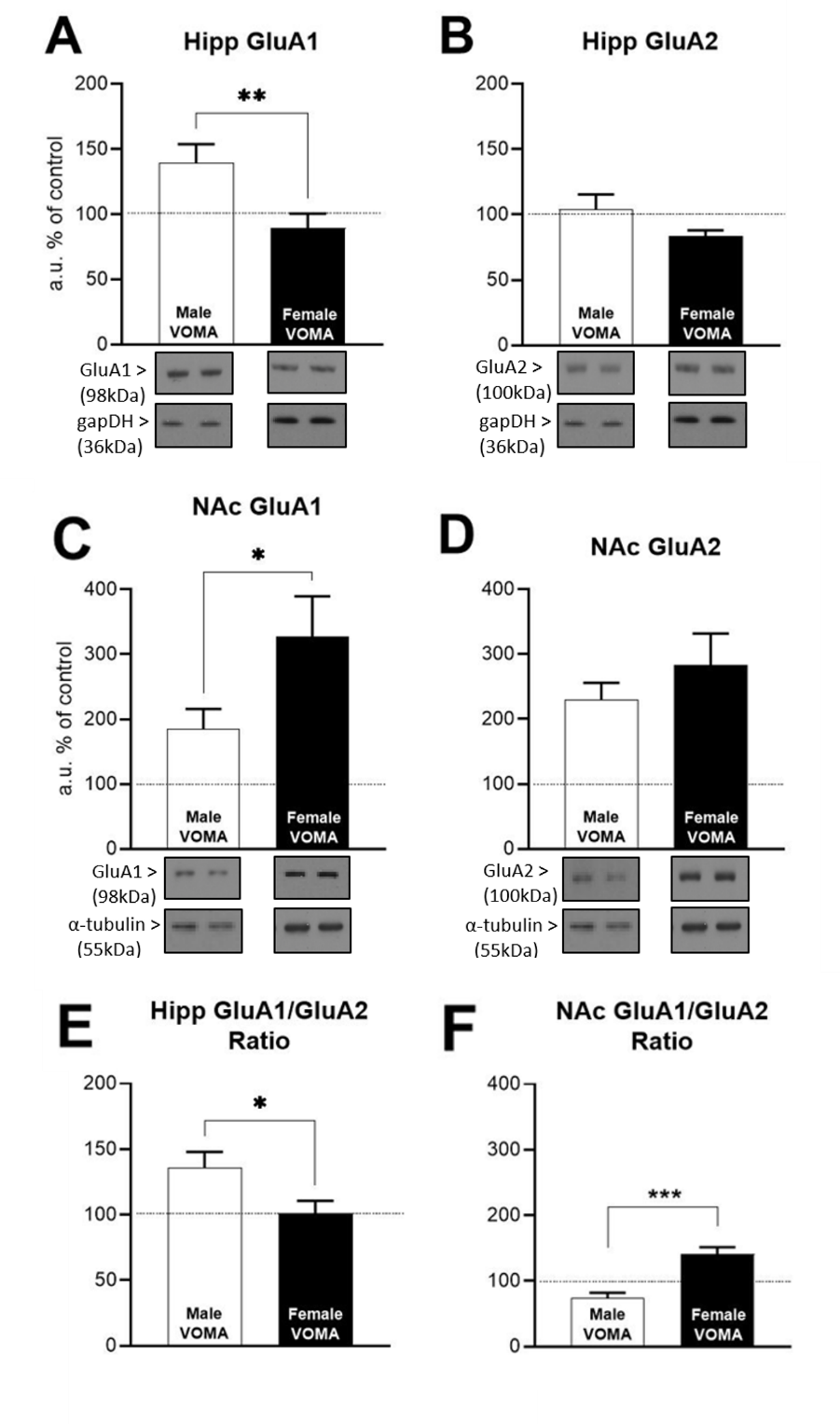


**Figure S1.** **Abstinence from the Escalation voluntary oral methamphetamine administration (VOMA) model produces female specific decreases in hippocampal GluA1 and increased GluA1 expression in the nucleus accumbens.** Hippocampal GluA1 (a) but not GluA2 expression (b) is significantly lower in female VOMA mice compared to male mice, with a lower ratio of GluA1/GluA2 expression in female mice only (e). Female VOMA mice demonstrate increased GluA1 (c) but not GluA2 (d) expression in the nucleus accumbens compared to male mice with an increase in the ratio of GluA1/GluA2 in female mice only (f). Representative blots are shown for two animals per group. Data shown here are a % control transformation of the data portrayed in Figure S2. VOMA: voluntary oral methamphetamine administration. * p< 0.05, ** p<0.01, ***p < 0.001.


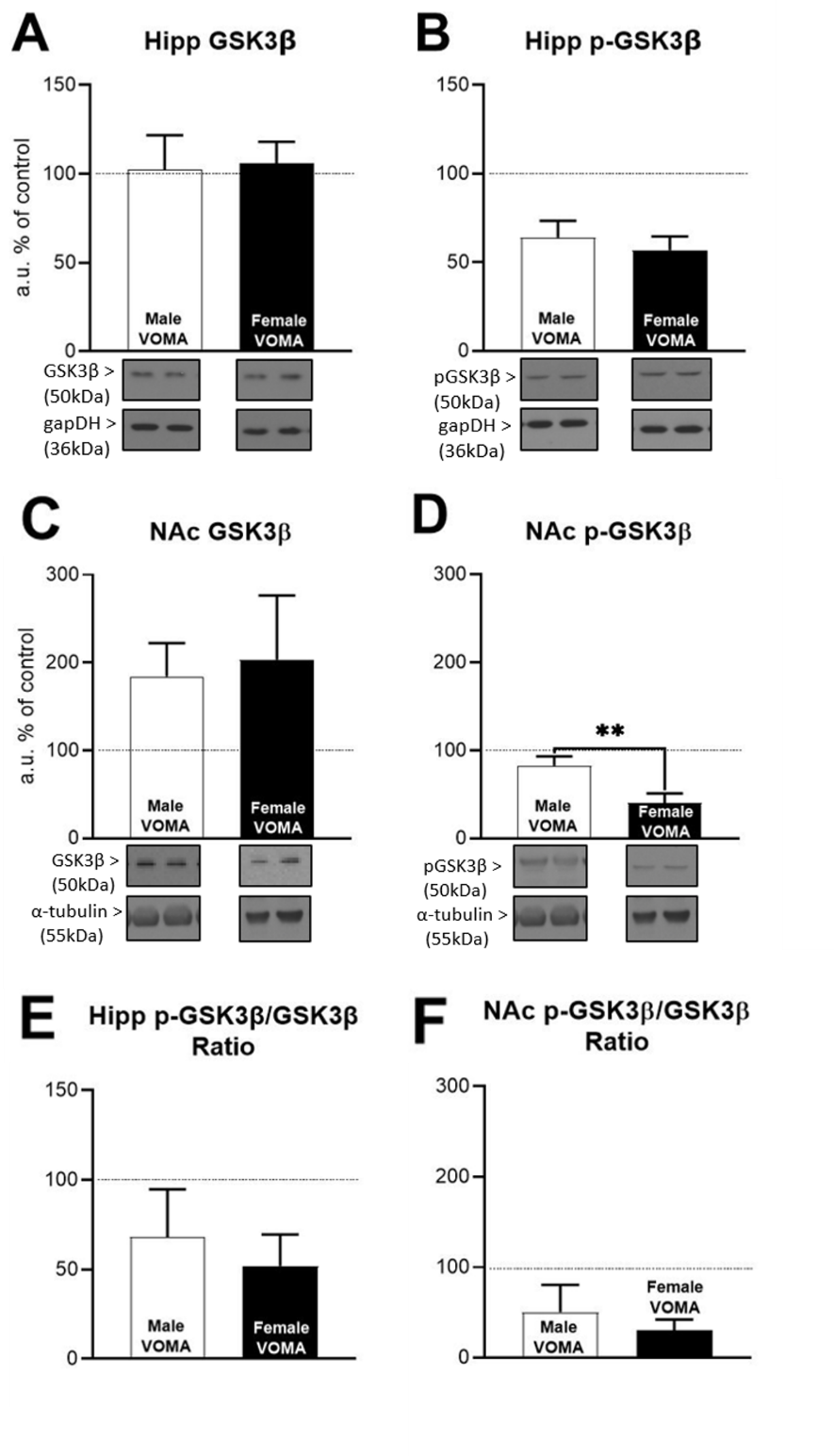


**Figure S2.** **Abstinence from VOMA produces a female specific decrease in p-GSK3β signaling in the nucleus accumbens.** Hippocampal GSK3β (a), p-GSK3β (b), and the ratio of p-GSK3β/GSK3β expression (e) were not significantly different between male and female VOMA mice following abstinence from chronic MA. Male and female VOMA mice do not demonstrate differences in GSK3β expression in the nucleus accumbens (c) but female VOMA mice decreased p-GSK3β expression (d) compared to male mice following abstinence. No differences in the ratio of p-GSK3β/GSK3β expression (f) in the nucleus following abstinence was observed between sexes. Representative blots are shown for two animals per group. Data shown here are a % control transformation of the data portrayed in Figure 3*.* VOMA: voluntary oral methamphetamine administration. ** p<0.01
