# Supplementary figures and images for: Synaptic remodeling of GluA1 and GluA2 expression in the nucleus accumbens promotes susceptibility to cognitive deficits concomitant with downstream GSK3β mediated neurotoxicity in female mice during abstinence from voluntary oral methamphetamine
Source: Addict Neurosci. Author manuscript; Available in PMC 2023 Dec 1. (PMC10569060; doi:10.1016/j.addicn.2023.100112)

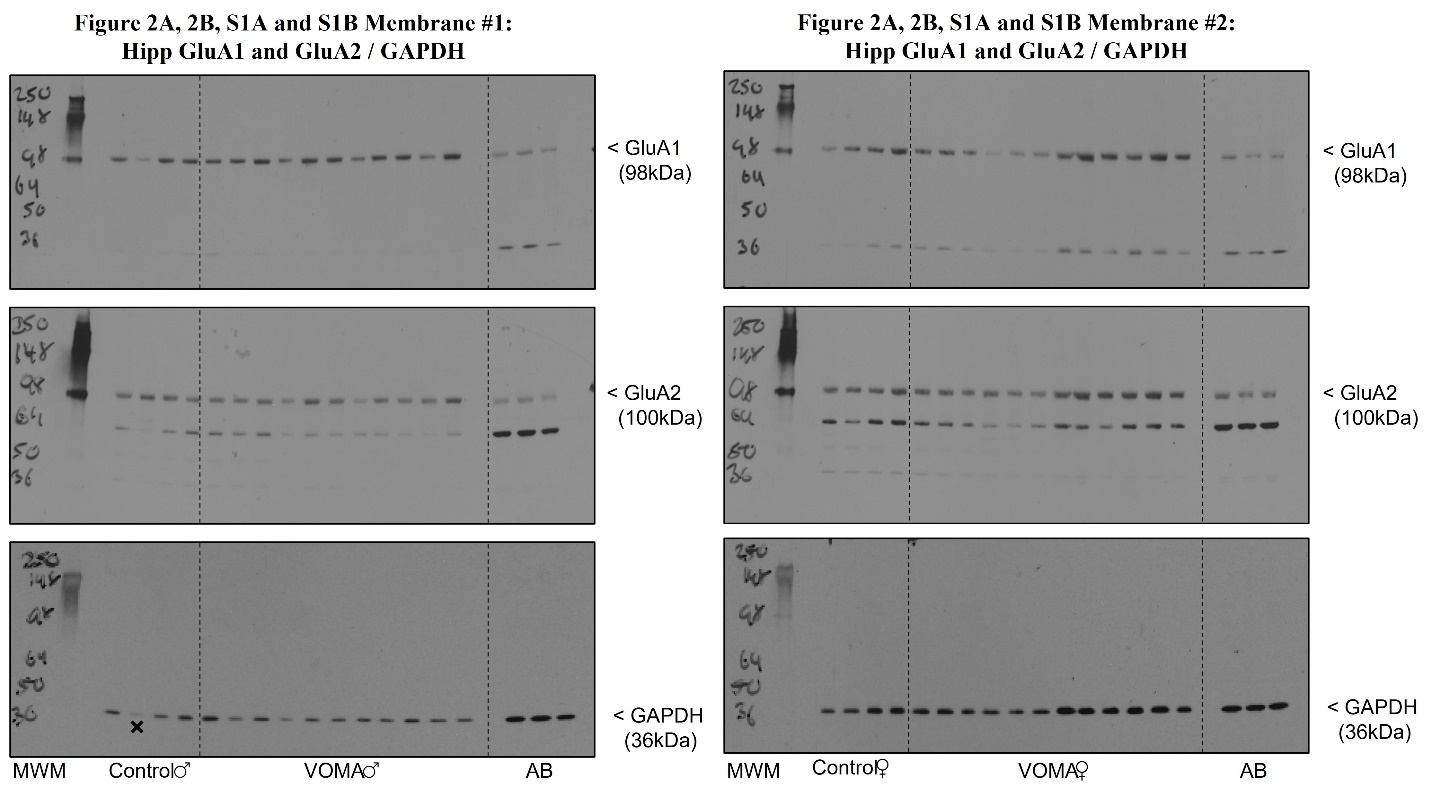


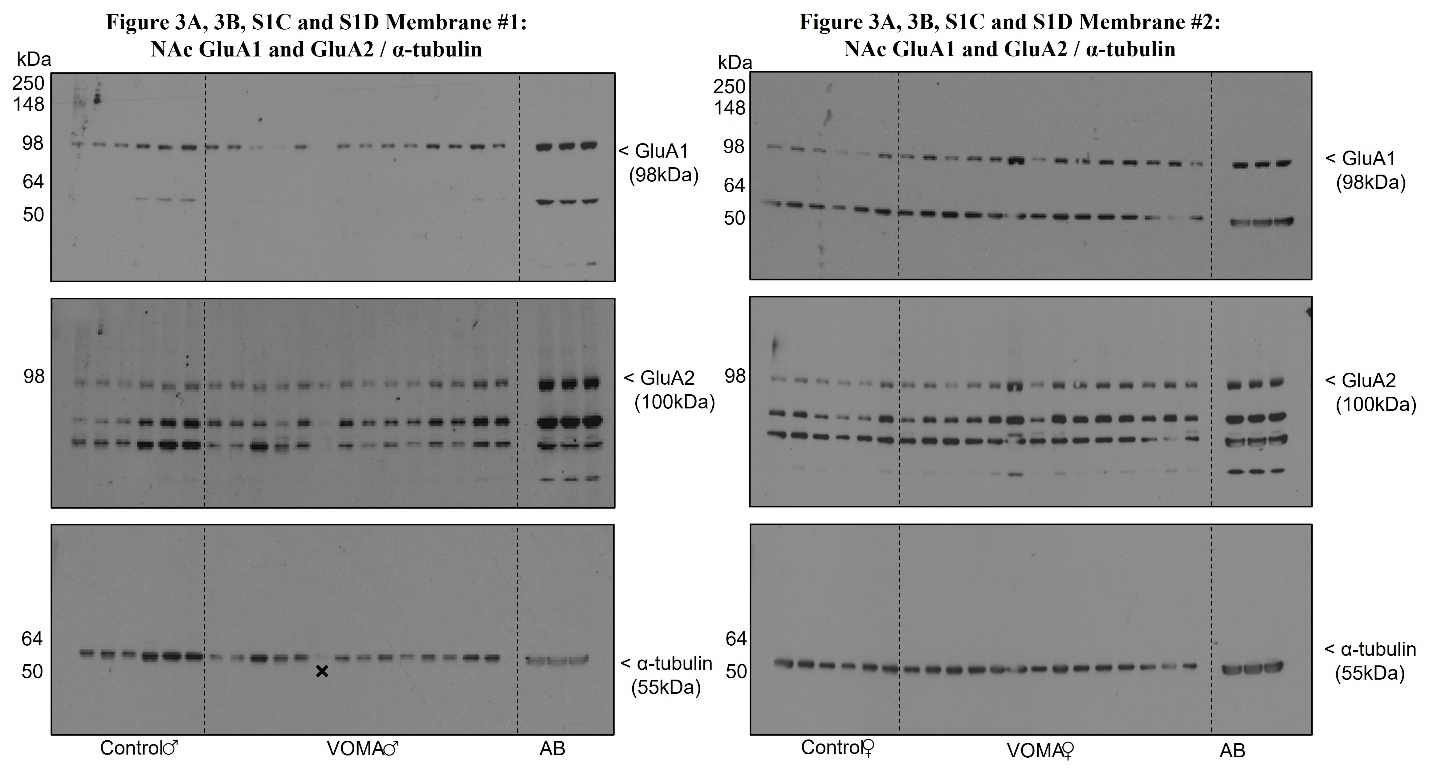


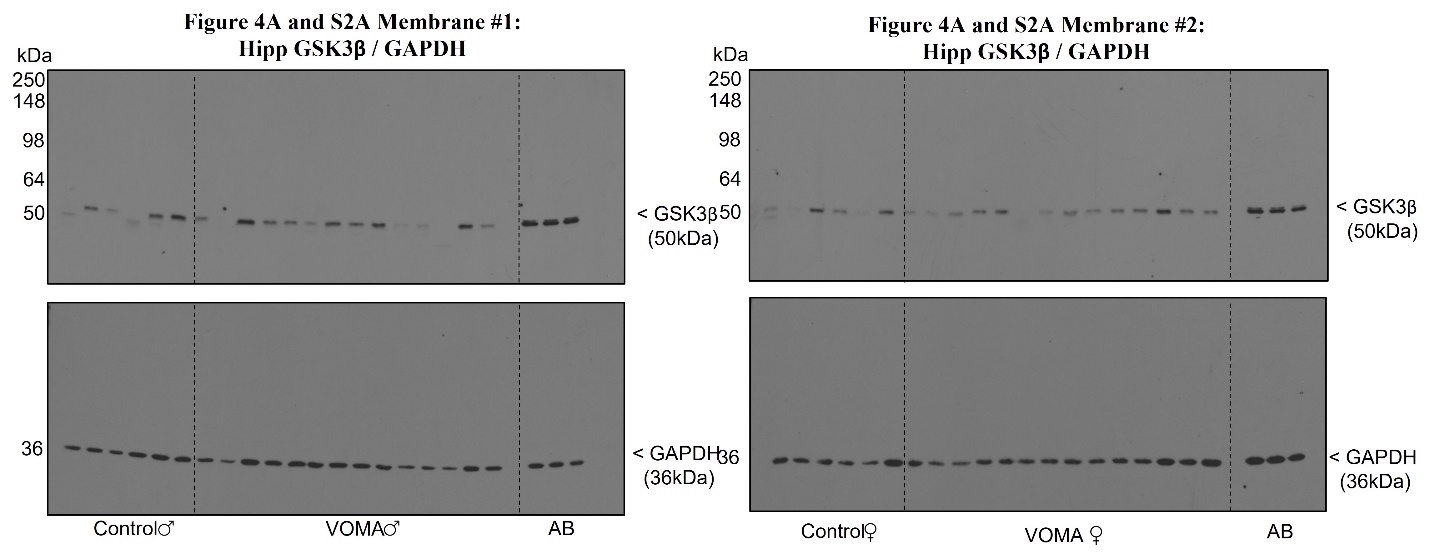


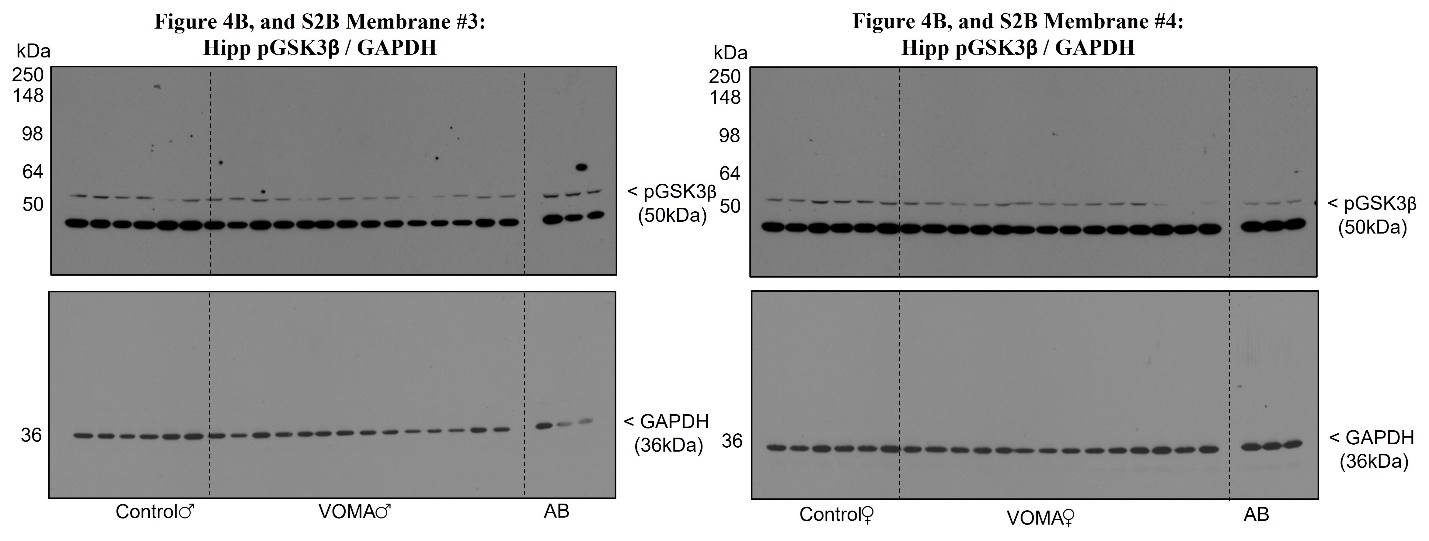


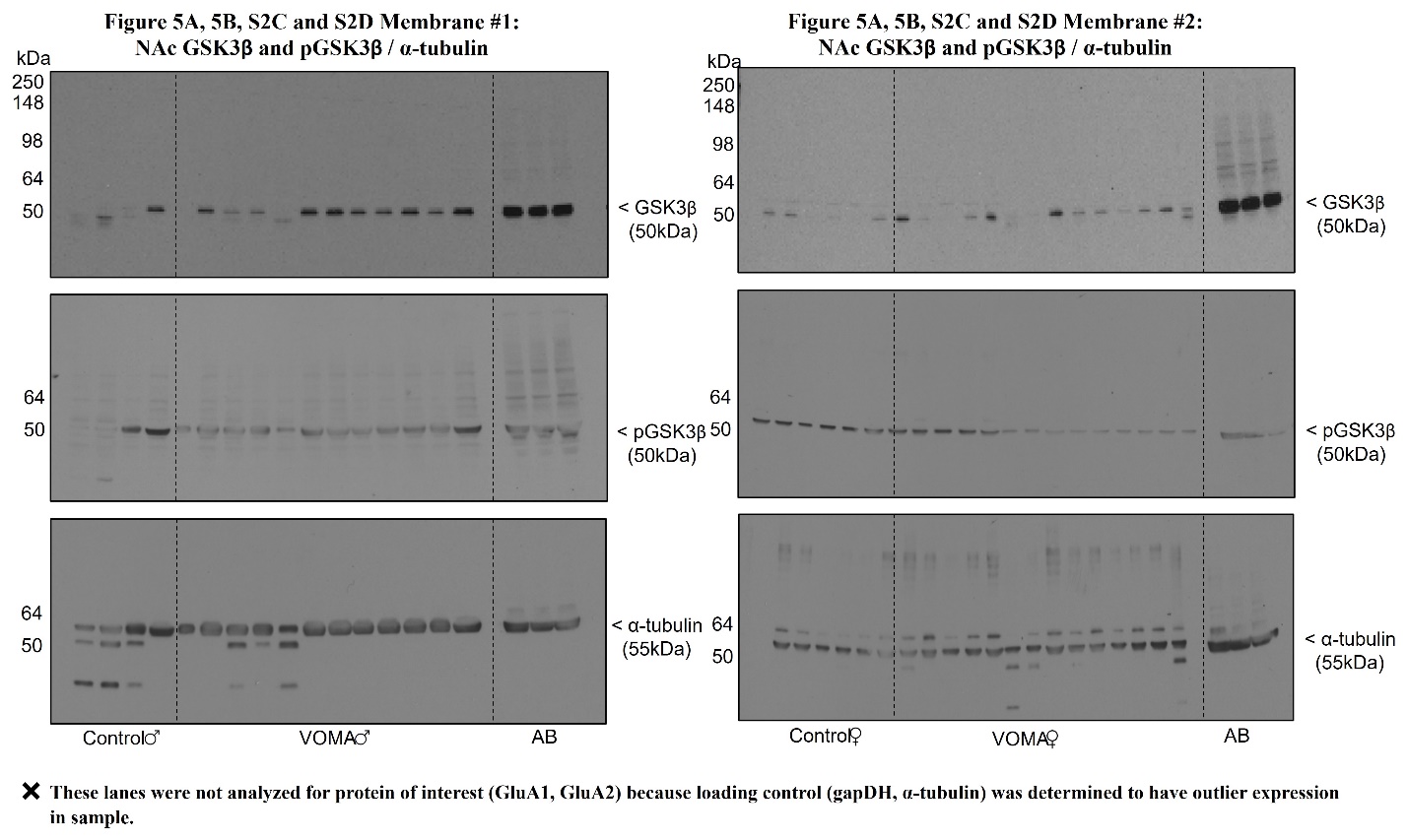

Supplement: Suppl1 [file NIHMS1925481-supplement-Suppl1.docx]
